# Relevance of selective neural stimulation with a multicontact cuff electrode using multicriteria analysis

**DOI:** 10.1371/journal.pone.0219079

**Published:** 2019-07-02

**Authors:** Mélissa Dali, Lucie William, Wafa Tigra, Hubert Taillades, Olivier Rossel, Christine Azevedo, David Guiraud

**Affiliations:** 1 INRIA, University of Montpellier, CNRS, Montpellier, France; 2 MXM, Sophia Antipolis, France; 3 Lab. Chirurgie Experimentale, Institut de Biologie, University of Montpellier, Montpellier, France; Georgia State University, UNITED STATES

## Abstract

Neural multicontact cuff electrodes have the potential to activate selectively different groups of muscles and offer more possibilities of electrical configurations compared to whole ring cuffs. Several previous studies explored multicontact electrodes with a limited set of configurations which were sorted using a selectivity index only. The objective of the present study is to classify a larger number of configurations, i.e. the way the current is spread over the 12 contacts of the cuff electrode, using additional criteria such as robustness (i.e. ability to maintain selectivity within a range of current amplitudes) and efficiency (i.e. electrical consumption of the considered multipolar configuration *versus* the electrical consumption of the reference whole-ring configuration). Experiments were performed on the sciatic nerve of 4 rabbits. Results indicated that the optimal configuration depends on the weights applied to selectivity, robustness and efficiency criteria. Tripolar transverse is the most robust configuration and the less efficient, whereas tripolar longitudinal ring is efficient but not robust. New configurations issued from a previous theoretical study we carried out such as steering current ring appears as good compromise between the 3 criteria.

## Introduction

**Fig 1:**
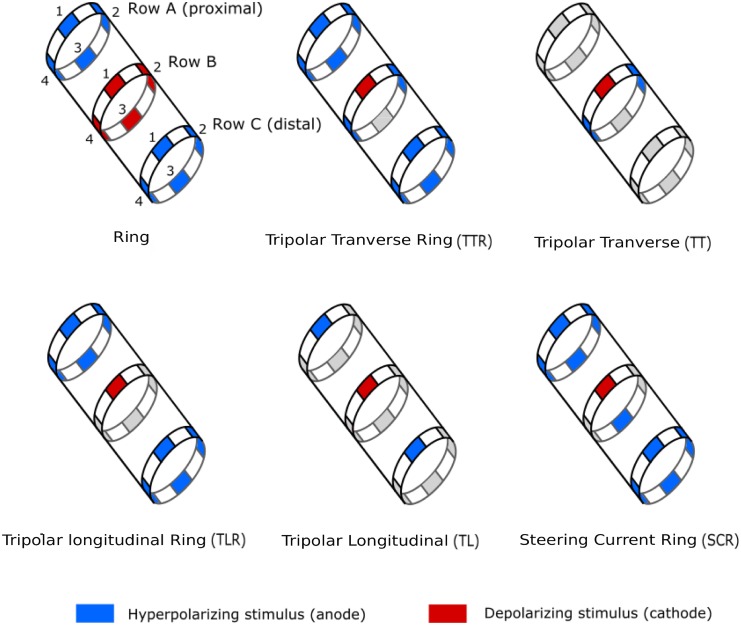
Six different configurations of the 12 contacts electrode were tested: Ring, tripolar transverse ring (TTR), tripolar transverse (TT), tripolar longitudinal ring (TLR), tripolar longitudinal (TL) and steering current ring (SCR). Each configuration is composed of a unique active site (cathode) on the central ring B (red) and several return path (anode, blue) for which the current ratio is imposed [9]. Only one electrode conformation is presented here (cathode position). The row A is proximal to the spinal cord whereas the row C is distal.

Functional neural electrical stimulation is a technique used to restore motor function in case of neurological deficits: the stimulation of peripheral nerves that innervate muscles can then generate muscle contraction and restore movements. The independent control of motor neuron pools is necessary to control different muscles or, at least, muscle groups. In invasive electrical stimulation, different electrode designs have been investigated with the aim of achieving selective muscle recruitment [1], i.e. activation of a subset of muscles innervated by the same nerve. Intraneural electrodes (inserted longitudinally or transversely in the nerve) were used to activate a single fascicle or very specific bundles of fibers [2–4] however the number of implants must be increased in order to access to the fascicles away from the electrode. Extraneural cuff electrodes wrapped around the nerve trunk limit invasiveness: multicontact cuff electrodes composed of multiple contact points were then developed to activate subpopulations of axons within the nerve. The most common designs used are the FINE cuff (flat interface nerve electrode)- that flatters the nerve trunk to better access the fascicles [5]—and the round cuff. With multicontact cuff electrodes, there are several possible combinations between active cathodes and anodes positions. Each combination constitutes one electrode configuration. The selectivity of several electrode configurations has been investigated through numerical simulations [6–9], and experiments were conducted on motor nerves such as the sciatic nerve [1, 10, 11]. In the study of Nielsen et al. [11] and Veraart et al. [10], a limited set of configurations were compared based on the selectivity principle, i.e. the ability of each configuration to activate the targeted axonal population. Veraart et al. [10] studied cat sciatic nerve branches activation using four electrode configurations. The objective was to selectively activate the Medial Gastrocnemius (MG), Soleus (Sol), Tibialis Anterior (TA) and Extension Digitorum Longus (EDL). They could activate antagonist (Sol and MG) and agonist (TA and EDL) selectively but not each muscle individually. Their major results indicated that selectivity was strongly dependent on the anodes’ repartition (hyperpolarizing current). Nielsen et al., [11] investigated the ability of three configurations (longitudinal tripolar ring, longitudinal tripolar and tripolar transverse Fig 1) to activate selectively three branches of the sciatic nerve. Results indicated that the tripolar transverse configuration was the most selective for small to medium branches but not for the large tibial nerve branch. These two studies focused on selectivity but neither the total needed injected charge nor the robustness of the selectivity by varying the current were investigated.

In the present paper, we present a comparative study between an extended set of six configurations using a multicontact cuff electrode implanted in the rabbit sciatic nerve. The selective activation of TA, Lateral Gastrocnemius (LG), MG and Sol as well as the activation of two antagonistic movements (plantar flexion and dorsiflexion) were studied. In addition to the selectivity criteria, clinically relevant parameters such as the robustness and the efficiency [9] were added to sort the configurations. They take into account the range of current that maintains selectivity (robustness) and the needed quantity of injected charge against a standard 3-ring cuff (efficiency). The main objective is to classify multipolar configurations according to the importance given to these three criteria and depending on the functional outcome targeted.

## Materials and methods

### Surgery

**Fig 2:**
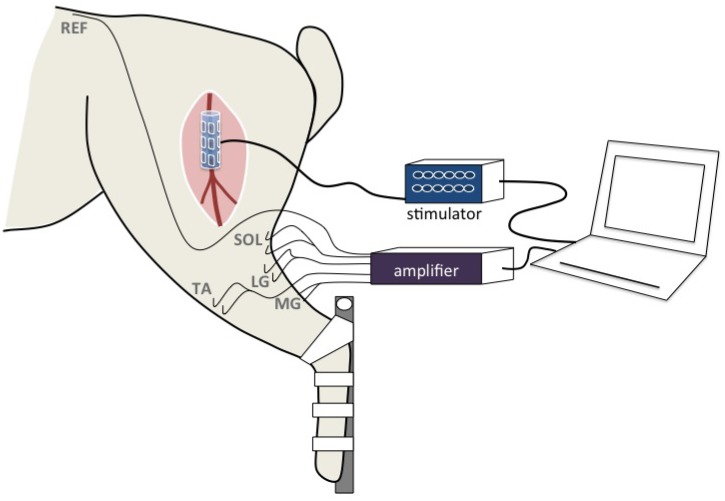
Setup description.

**Fig 3:**
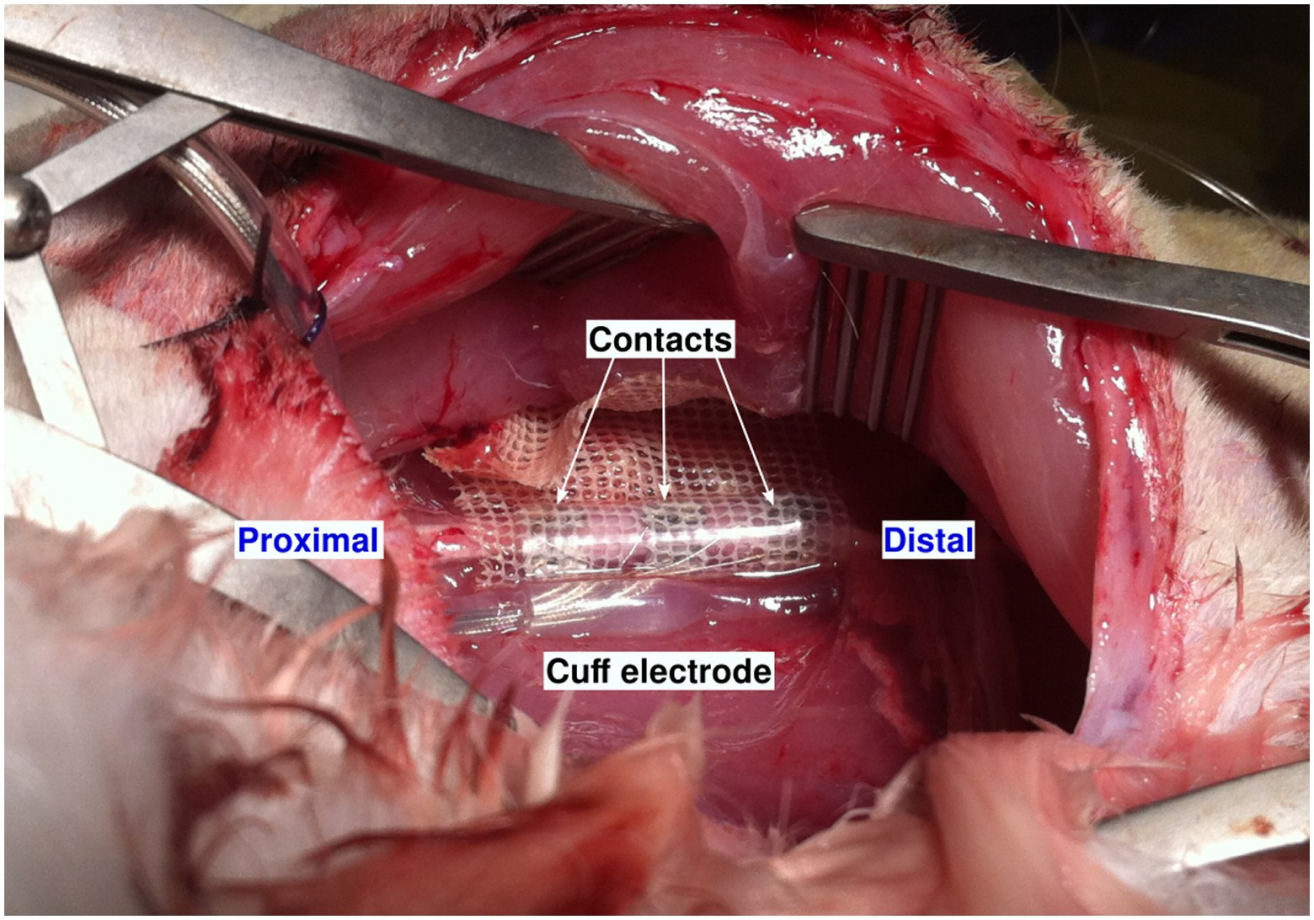
Cuff electrode placed around the nerve.

Four adult male New Zealand rabbits weighing 3.7±0.38 kg were used in this study. All procedures related to this study were approved by the Animal Care and Use Committee Languedoc-Roussillon (reference: # CEEA-LR-12084). The animals were initially anesthetized with an injection of 53.19 mg/kg Ketamine, 2.66 mg/kg Xylazine and 0.53 mg/kg Acepromazine. They were perfused with isotonic sodium chloride solution. Anesthesia was maintained with additional injections of the half dose 30 minutes later. Same injection was then delivered on demand (approximately every hour) until the end of the experimentation when the rabbits were euthanized with an overdose of barbiturate. The left foot of the rabbit was fixed to a mechanical frame using tape. The sciatic nerve was exposed after dissecting the biceps femoris (Fig 2). Then the electrode was placed around the nerve and gently sutured (Fig 3).

### Stimulation

The stimulator R&Stim 12 (Axonic, Vallauris, France) is based on the work of [12], and was connected to a laptop and controlled by a software developed for multicontact electrode control. The software allows not only to control the stimulation parameters (waveform, pulse width, intensity, frequency) but also the spreading of the current over the 12 contacts through the programming of ratios (from 1/15 to 15/15) and the polarity (cathodic / anodic) of each contact. A 12 contacts cuff electrode (Axonic, Vallauris, France) was used for the experiments (Fig 2). The total length of the cuff was 20 mm. The cuff electrode contained three rows of four 4 mm x 2 mm (8 mm^2^) contacts separated with 1.5 mm intervals. The distance between rows was of 3 mm and the distance between the external silicon edge and the contacts 1 mm. The inner diameter of the cuff was 2 mm. Biphasic constant current rectangular balanced pulse of 250 *μ*s pulse-width and 100 *μ*s interstim [13] with a 4 Hz frequency were delivered by the stimulator. The intensity was modulated through steps of different amplitudes depending on the useful range on each rabbit (Table 1); besides, tripolar transverse configuration needs much more current than others and thus has a different current step.

**Table 1:** Summary of the current intensities used for each rabbit.

Rabbit	Configurations	Intensity (*μ*A)
Rabbit 1	Ring—TTR—TLR—TL—SCR	262.5, 300, 337.5, 375, 412.5, 450, 487.5, 525, 562.5, 600, 637.5, 675, 712.5, 750, 787.5, 825, 862.5, 900, 937.5, 1012.5, 1050
	TT	300, 375, 450, 600, 750, 900, 1050, 1200, 1350, 1500, 1800
Rabbit 2	Ring—TTR—TLR—TL—SCR	37.5, 75, 112.5, 150, 187.5, 225, 300, 375, 450, 600, 750, 950
	TT	75, 112.5, 150, 225, 300, 375, 450, 600, 750, 1050, 1350, 1800
Rabbit 3	Ring—TTR—TLR—TL—SCR	52.5, 74, 112.5, 150, 187.5, 225, 300, 375, 450, 525, 600, 675, 750, 825
	TT	150, 187.5, 225, 300, 375, 450, 600, 750, 900, 1050, 1125, 1500, 1800
Rabbit 4	Ring—TTR—TLR—TL—SCR	37.5, 75, 112.5, 150, 187.5, 225, 300, 375, 450, 600, 750, 950
	TT	150, 187.5, 225, 300, 375, 450, 600, 750, 900, 1050, 1200, 1500, 1800

### Electrode configuration and conformation

A multicontact cuff electrode configuration is defined as the current repartition over the different contacts whereas the electrode conformation corresponds to the position of the cathode on the row (4 electrode conformations available per row, except for Ring). The convention used is that the cathode depolarized the nerve fiber, and the anode hyperpolarized the nerve fiber. Six configurations corresponding to different arrangements of active contacts were explored: Ring, tripolar transverse ring (TTR), tripolar transverse (TT), tripolar longitudinal ring (TLR), tripolar longitudinal (TL) and steering current ring (SCR) (Fig 1). The last five configurations were initially found through the simulation of a generic nerve model [9] as optimal solutions of different cost functions; the cost functions were defined as weighted sums of the 3 criteria. For all the configurations, the cathode was placed on the central row (row B). Row A was proximal to the spinal cord whereas row C was distal. The Ring configuration (whole ring) was added as the reference for benchmarking. The Ring configuration is considered not selective as it stimulates the whole nerve trunk. All the configurations were scanned in the same order for all the animals. The stimulation was repeated 9 times per conformation. The Ring configuration was tested twice at the beginning and at the end of the scan to assess the change in muscle recruitment. Electrode integrity was checked before and after the surgery to ensure that no failure occurred during the experiment.

### Recording

Bipolar wire hook recording electrodes, made of Teflon-coated silver wires (75 μm) were inserted in the 4 studied muscles (TA, LG, MG, Sol). A common ground needle electrode was inserted under the back skin. TA induces dorsiflexion of the foot. MG, LG and Sol are agonists inducing plantar flexion of the foot.

These recording electrodes were connected to a differential amplifier g.BSamp (Gtec, Austria) with gain 1,000 and bandpass filtered (0.5 Hz-1 kHz). The 50 Hz Notch filter was set on. Electromyography (EMG) signals were sampled at 10 kHz (PowerLab, ADInstrument). Compound Muscular Action Potentials (CMAPs) were collected in bipolar mode.

### Data analysis

#### Indexes

Data analysis was performed off-line using MATLAB (Mathworks). Root mean square (RMS) values of compound muscular action potential (CMAP) were normalized to the maximum RMS value of CMAP to express the response as a fraction of full muscle activation (recruitment *r*). For each stimulation configuration (*conf*), cathode conformation (*cath*) and stimulation intensity (*I*), the selectivity index (*SI*) was calculated as the recruitment *r*_*conf*,*cath*,*m*_ of the considered muscle (*m*) divided by the sum of the recruitment of all 4 muscles as follows:

$$\begin{array}{c} SI_{conf,cath,m}=\frac{r_{conf,cath,m}\left(I\right)}{\sum_{j=1}^{4}r_{conf,cath,j}\left(I\right)} \end{array}$$ (1)

**Fig 4:**
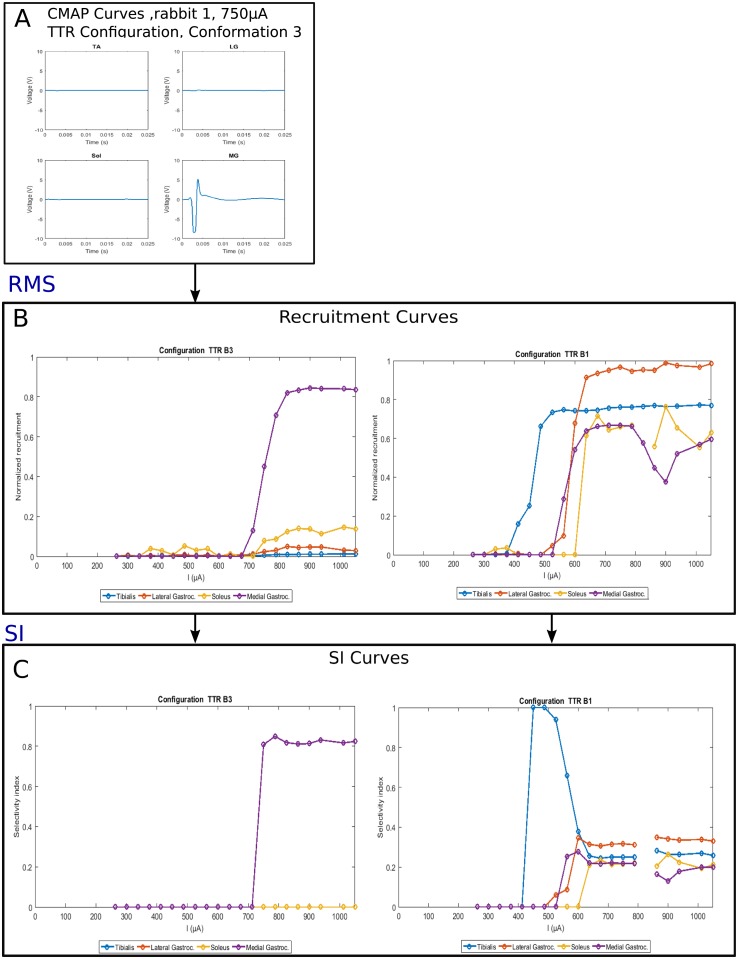
Different steps of the signal processing illustrated through data from Rabbit 1 with TTR configuration. **A** example of CMAPs collected for each muscle, configuration TTR(B3). **B** Recruitment curves obtained after the computation of RMS: TTR(B3) (left) and TTR(B1) (right). **C** Selectivity curves obtained for TTR(B3) (left) and TTR(B1) (right). Missing point corresponds to data that could not be recorded.

The whole process is described on Fig 4.

We defined a selective and functional criteria, the SIR, based on Nielsen et al. [11]. The SIR is valid if, for a single muscle *m*, a given configuration *conf* and a conformation *cath*, the SI index and the recruitment are above 70%.

$$\begin{array}{c} SIR=true\;if\;SI_{conf,cath,m}>70\%\;\&\;r_{conf,cath,m}>70\% \end{array}$$ (2)

If for a given muscle, the double criteria was not achieved, we computed the maximum *SI* when the movement was sufficiently functional, which means that the recruitment was above 20%. Else-wise, if the recruitment was insufficient to induce a functional movement, *SI* was set to 0.

For the configurations meeting the criteria, two other indexes were calculated: the robustness and the efficiency is defined in [9]. The robustness (*Rob*) quantifies the ability of the configuration to maintain *SI* when the current changes by ±50% (respectively *SI*_0.5_ and *SI*_1.5_) as follows:

$$\begin{array}{c} Rob=1-\frac{|SI-SI_{0.5}|+|SI-SI_{1.5}|}{2} \end{array}$$ (3)

The efficiency (*Eff*) compares the delivered charge of studied configuration to the whole Ring configuration:

$$\begin{array}{c} Eff=\frac{I_{stim_{ring}}}{I_{stim_{ring}}+I_{stim}} \end{array}$$ (4)

To compare the configurations between each other according the importance given to *SI*, *Rob* and *Eff*, we defined different weight’s sets *w*_*SI*_, *w*_*Rob*_, *w*_*Eff*_ (Table 2). For instance, the set *w*_*SI*_ = 1/3, *w*_*Rob*_ = 1/3, *w*_*Eff*_ = 1/3 corresponds to the best compromise between selectivity, robustness and efficiency, the set *w*_*SI*_ = 2/3, *w*_*Rob*_ = 1/3, *w*_*Eff*_ = 0 corresponds to a compromise that favors more selectivity than robustness, without taking account the efficiency criteria. The cost function is defined by a quadratic form (Eq 5). The weights make it possible to modulate the importance of each criterion. The configuration that best fitted the weights set corresponded to the one that had the minimum weighted distance. This method enabled the sorting of the responses to stimulation, not only based on the achieved selectivity but also on the importance given to the robustness and the efficiency. It could be relevant for clinical applications to select a compromise between the different indexes instead of the best *SI*.

$$\begin{array}{c} \begin{array}{c} D_{\left(w_{SI},w_{eff},w_{rob}\right)}=({\left(w_{SI}*\left(1-SI_{conf,cath,m}\right)\right)}^{2}+\\ {\left(w_{eff}*\left(1-Eff_{conf,cath,m}\right)\right)}^{2}+{\left(w_{rob}*\left(1-Rob_{conf,cath,m}\right)\right)}^{2}{))}^{1/2} \end{array} \end{array}$$ (5)

**Table 2:** Weight’s set used to compute the weighted cost function *D*.

*w*_*SI*_	*w*_*Rob*_	*w*_*Eff*_
1/3	1/3	1/3
1/3	0	2/3
2/3	0	1/3
1/3	2/3	0
2/3	1/3	0
1	0	0

## Results

### Recruitment and maximum SI index

All the configurations tested reached a minimum of functional movement (*recruitment* > 20%) whatever the animal and muscle. Table 3 represents the configurations that reached maximum SI for each animal and each muscle. Among the 18 optimal configurations presented, SI was greater than 0.34. 9/18 configurations had SI superior to 0.7 and 5/18 reached the *SIR* criteria (Eq 2) (Table 3, bold). Concerning animal 1, for LG and Sol muscles, two configurations had the same SI index but different current thresholds and recruitment levels. Finally, the *SIR* criteria was never reached for LG and Sol.

**Table 3:** Maximum SI obtained for each rabbit, configuration and muscle. Bold numbers indicate that SIR is fulfilled.

Rabbit	Muscle	Configuration (Conformation)	SI	Recruitment	Intensity (*μ*A) ± step
Rabbit 1	TA	**SCR (B4)**	**0.97**	**71.9%**	1012.5 (± 37.5)
	LG	TTR (B2)	0.57	68.5%	562.5 (± 37.5)
		SCR (B2)		60.3%	450 (± 37.5)
	Sol	SCR (B2)	0.35	73.9%	675 (± 37.5)
		TT (B2)		77.5%	1500 (± 300)
	MG	**TL (B3)**	**0.83**	**83.7%**	562.5(± 37.5)
Rabbit 2	TA	**SCR (B4)**	**0.94**	**70.3%**	600 (± 150)
	LG	TT (B1)	0.37	40.9%	375 (± 75)
	Sol	TLR (B1)	0.39	79.2%	300 (± 75)
	MG	TLR (B3)	0.81	22.9%	187.5 (± 37.5)
Rabbit 3	TA	TTR (B1)	0.95	27.2%	600 (± 150)
	LG	TL (B2)	0.38	78.1%	675 (± 75)
	Sol	TT (B4)	0.34	72.1%	900 (± 150)
	MG	TTR (B4)	0.94	57.3%	450 (± 150)
Rabbit 4	TA	**TLR (B4)**	**0.76**	**86.9%**	187.5 (± 37.5)
	LG	TTR (B1)	0.36	81.2%	600 (± 150)
	Sol	TLR (B3)	0.92	27.4%	112.5 (± 37.5)
	MG	**TTR (B2)**	**0.82**	**92.0%**	450 (± 150)

### Optimal configurations

The *Rob* and *Eff* indexes were determined for each rabbit, muscles and configurations. Values obtained for the rabbit 1 are presented in Table 4, the results for the others rabbits are presented in Supporting information (S1 to S3 Tables). This table showed that the optimal configuration and conformation that gave the maximum SI may not provide an efficient or robust solution. The results should be further sorted using the weighted global criterion: cost function *D* was then computed to determine optimal configurations according to the weight sets.

**Table 4:** Rabbit 1. SI, Rob and Eff. Indexes are presented for each muscle and configuration (with optimal cathode conformation for each muscle).

Muscle	Configuration (Conformation)	SI	Rob	Eff	Recruitment	Intensity (*μ*A) ± step
TA	TTR (B1)	0.94	0.48	0.46	73.4%	525 (± 37.5)
	TLR (B4)	0.77	0.52	0.44	72%	562.5 (± 37.5)
	TL (B4)	0.93	0.39	0.44	73.9%	562.5 (± 37.5)
	SCR (B4)	0.97	0.93	0.31	71.9%	1012.5 (± 37.5)
	TT (B1)	0.94	0.22	0.3	89.7%	1050 (± 150)
LG	TTR (B2)	0.57	0.71	0.56	68.5%	562.5 (± 37.5)
	TLR (B2)	0.45	0.71	0.66	87.9%	365 (± 37.5)
	TL (B2)	0.45	0.7	0.66	83.7%	375 (± 37.5)
	SCR (B2)	0.57	0.88	0.61	60.3%	450 (± 37.5)
	TT (B2)	0.4	0.93	0.54	89.1%	1500 (± 300)
Sol	TTR (B1)	0.26	0.85	0.38	76.4%	900 (± 37.5)
	TLR (B1)	0.31	0.95	0.38	100%	900 (± 37.5)
	TL (B2)	0.28	0.73	0.6	52%	375 (± 37.5)
	SCR (B2)	0.35	0.72	0.45	73.9%	675 (± 37.5)
	TT (B2)	0.35	0.82	0.43	77.5%	1500 (± 300)
MG	TTR (B3)	0.81	0.66	0.31	84.5%	900 (± 37.5)
	TLR (B3)	0.77	0.54	0.42	83.5%	562.5 (± 37.5)
	TL (B3)	0.83	0.47	0.42	83.7%	562.5 (± 37.5)
	SCR (B3)	0.8	0.73	0.31	85.2%	937.5 (± 37.5)
	TT (B2)	0.46	0.82	0.19	40.6%	1350 (± 150)

The Table 5 shows the frequency of the optimal configurations obtained according to the animal, the muscle or the weighted cost function *D*. The case of multiple solutions (i.e. several configurations give the same value of the cost function *D*), have been included on the table. Pearson’s Chi squared test for independence was performed. The optimal configuration depends on the animal (*p*-*value* < 0.01) and on the cost function (*p*-*value* = 0.04) but was independent of the muscle (*p*-*value* = 0.75). For the Rabbit 1, the optimal configuration which appeared the most was the SCR, for the Rabbit 2 and 4 the TLR and for the Rabbit 3 the TT. The results show that, **selective and robust configurations were preferentially TT and eventually TTR or SCR**. Whereas **selective and efficient configurations were preferentially TLR, TL and eventually SCR**. In the case where efficiency has been favored over selectivity (*w*_*SI*_ = 1/3,*w*_*Rob*_, *w*_*Eff*_ = 2/3), multiple results appeared for LG (rabbit 1) and TA (rabbit 2): TL and TLR. **Configurations combining selectivity, robustness and efficiency are preferentially SCR, eventually TL**. It should be compared to the use of SI alone for which the best configurations are not well determined. Multiple results for rabbit 1 also appeared in this case: TTR and SCR for the LG muscle, TT and SCR for the Sol muscle.

**Table 5:** Configuration occurrence as the optimal solution according to i) animal, ii) muscle, iii) weighted cost function *D*. “Multiple” means that more than one configuration was found to be optimal (equal cost function *D*).

Configurations versus				TTR	TLR	TL	SCR	TT	Multiple
**Animal**	**Rabbit 1**			3	2	6	13	3	5
	**Rabbit 2**			3	10	8	2	6	3
	**Rabbit 3**			6	4	7	5	8	2
	**Rabbit 4**			6	10	2	7	6	1
**Muscle**	**TA**			6	3	3	7	4	1
	**LG**			5	4	5	6	2	2
	**Sol**			1	6	4	6	6	1
	**MG**			4	7	5	4	4	0
**Cost function *D***	*w*_*SI*_	*w*_*Rob*_	*w*_*Eff*_						
	**1/3**	**0**	**2/3**	1	4	6	3	0	2
	**1/3**	**2/3**	**0**	4	2	1	3	6	0
	**1**	**0**	**0**	4	4	2	2	2	2
	**2/3**	**0**	**1/3**	2	6	3	4	1	0
	**2/3**	**1/3**	**0**	3	2	1	4	6	0
	**1/3**	**1/3**	**1/3**	2	2	4	7	1	0

### Functional results

In order to analyze the results from a functional viewpoint, the 4 muscles were clustered into 2 groups: the first one, named flexor, is limited to the agonist muscle of the flexion (TA) and the second, named extensor, gathers the agonist muscles of the extension (MG, LG and Sol). The recruitment of the extensor group was computed as the average recruitment of these 3 muscles. Then *SI*, *Rob* and *Eff* were computed accordingly.

In the 4 animals, the 2 groups of muscles were activated respecting the double criteria *SIR* (Table 6), except for rabbit 3 for the flexor group due to a low recruitment rate (see Annexe). Flexor groups were activated respecting *SIR* with the configurations TTR (2 among 4 rabbits), TLR (2/4), TL (1/4), SCR (3/4) and TT (2/4): in all these configurations the cathode was always placed at the same location or just close to. The extensor was activated respecting *SIR* with the configurations TTR (2/4), TL (1/4), SCR (1/4) and TT (2/4): the cathode was always at the same location or just the one close to. Furthermore cathode position for flexor and extensor position were never the same further demonstrating the fascicle organization and the relevance of such an approach.

**Table 6:** Configurations that reach the *SIR* criteria for the antagonist and agonist groups.

Rabbit	Movement	Configuration (conformation)	*SI*	*Rob*	*Eff*	Recruitment	Intensity
Rabbit 1	Flexor	TTR (B1)	0.85	0.83	0.46	75%	562.5 (±37.5)
		TLR (B4)	0.91	0.64	0.46	72%	562.5 (±37.5)
		TL (B4)	0.98	0.61	0.46	74%	562.5 (±37.5)
		SCR (B4)	0.99	1.00	0.33	72%	1012.5 (±75)
		TT (B1)	0.98	0.34	0.32	90%	1050 (±150)
	Extensor	TT (B2)	0.96	0.98	0.28	78%	1800 (±300)
Rabbit 2	Flexor	TTR (B4)	0.97	0.99	0.43	80%	600 (±150)
		SCR (B4)	0.98	0.59	0.43	70%	600 (±150)
	Extensor	TTR(B1)	0.92	0.81	0.40	86%	450 (±150)
Rabbit 3	Flexor	No configuration achieved the SIR					
	Extensor	TT (B4)	0.72	0.89	0.40	77%	1125 (±375)
Rabbit 4	Flexor	TLR (B4)	0.91	0.42	0.62	87%	187.5 (±37.5)
		SCR (B4)	0.88	0.58	0.62	73%	187.5 (±37.5)
		TT (B4)	0.87	0.99	0.20	92%	1200 (±150)
	Extensor	TTR (B1)	0.72	0.91	0.43	86%	950 (±200)
		TLR (B2)	0.74	0.82	0.67	75%	375 (±75)
		SCR (B2)	0.9	0.94	0.50	73%	750 (±200)

## Discussion

In this paper a large set of selective stimulation configurations have been applied to the sciatic nerve of rabbit and were compared between each other based on a multicriteria weighted cost function. We showed that it was possible to elicit a selective activation of individual muscles through a single multicontact cuff electrode by piloting the cathode position and the anode active spreading of the current over the different remaining contacts. In particular the TA muscle could always be activated independently provoking the dorsiflexion of the foot. On the other hand a global extension of the foot was also possible in 3 rabbits out of 4: both antagonist movements are reachable with more than 70% of recruitment. For finer extension movement (Sol, MG, LG), *SIR* was only obtained for the MG muscle in 2 rabbits. However, the extension movement could be qualitatively changed depending on the proportion of each muscle recruitment.

Weighting the indexes offers the possibility to sort optimal solutions depending on the compromise we look for. In our previous paper, [9], TTR was the best compromise between the 3 criteria. In this study, we found experimentally that SCR was more appropriate as a compromise between the 3 criteria. Indeed, the optimization results depend not only on the definition of the cost function (weights and criteria) but also on the size of the nerve and the size of the targeted zone i.e. the targeted fascicle. Besides, the results were similar between model and experiments for TT and TLR. It shows that the way to find optimal solution through modeling needs to know a rough estimation of the nerve diameter and the sizes of fascicles added to the generic nerve model we already developed. Currently the detailed histology is not needed.

TT, a well described configuration in the literature, is the most selective and robust but less efficient as it requires high currents. TTR/SCR configurations appeared to be a good option between robustness and selectivity with lower current consumption than TT configuration. TLR and TL were found to be a compromise between efficiency and selectivity.

A clinical application would consider selective configurations from the most efficient and robust such as SCR/TLR/TTR and then try TT if the first set is not selective enough. Using 12-contacts cuff limits the invasiveness of the surgical procedure. One electrode could elicit activation of nerves muscles without increasing the number of implants in each nerve branches. Even if we could change the activation area by changing the configuration, the muscle selectivity remains dependent on electrode geometry such as the number of contacts.

For a given targeted muscle, the optimal configurations / conformations always refer to adjacent cathodes i.e. (Rabbit 1 Flexor SCR(B4) and TT(B1)). It further confirms the spatial organization of the nerve. Moreover, when 2 cathodes elicits almost the same selectivity, we may hypothesize that the underlying fascicle is strong and that these 2 poles target sets of motor units of the same muscles, with some overlap. This property may be used to further enhance the stimulation schedule by alternating between both cathode thus leading to limiting fatigue.

Finally, the quantitative results further confirm that the previous theoretical and simulation search of the optima on a generic nerve without *a priori* knowledge on the fascicle organization is relevant and provide configuration with expected performances providing we take into account the nerve diameter and a guess about the size of the targeted zone The results show that the effective optimal configuration is animal and cost function dependant. It is not surprising regarding the cost function, and concerning the animal it means that fascicle arrangement and probably their sizes are different so the solution. Our approach was designed to optimize several criteria but also several size and position of fascicle. it proves that one solution is not optimal for all cases but our set is well suited to fit all the encountered situations.

## Conclusion

This paper provides a novel method to explore and rank selective configurations using a multicontact cuff electrode to elicit selective movements from a single nerve. It relies on a stimulator capable of providing a constant ratio between contacts and a set of predetermined configurations obtained by mathematical modeling and simulation. The next step will be to transpose the concept to a human nerve to elicit motor control with a minimum set of implanted electrodes.

## Supporting information

S1 Table Rabbit 2. SI, Rob and Eff. Indexes are presented for each muscle and configuration (with optimal cathode conformation for each muscle).
(PDF)

S2 Table Rabbit 3. SI, Rob and Eff. Indexes are presented for each muscle and configuration (with optimal cathode conformation for each muscle).
(PDF)

S3 Table Rabbit 4. SI, Rob and Eff. Indexes are presented for each muscle and configuration (with optimal cathode conformation for each muscle).
(PDF)

## Abbreviations

CMAPs: Compound Muscular Action Potentials
Eff: Efficiency
EMG: Electromyography
LG: Lateral Gastrocnemius
MG: Medial Gastrocnemius
RMS: Root Mean Square
Rob: Robustness
SCR: Steering Current Ring
SI: Selectivity Index
Sol: Soleus
TA: Tibialis Anterior
TL: Tripolar Longitudinal
TLR: Tripolar Longitudinal Ring
TT: Tripolar Transverse
TTR: Tripolar Transverse Ring
