# Supplementary material for: Relevance of selective neural stimulation with a multicontact cuff electrode using multicriteria analysis
Source: PLoS One. 2019 Jul 2;14(7):e0219079. doi: 10.1371/journal.pone.0219079 (PMC6605667; doi:10.1371/journal.pone.0219079)
Supplement: S1 Table — Indexes are presented for each muscle and configuration (with optimal cathode conformation for each muscle). (PDF) [file pone.0219079.s001.pdf]

S1 Table

Muscle	Configuration (Conformation)	SI	Rob	Eff	Recruitment	Intensity (μA) $\pm$ step
TA	TTR (B4)	0.91	0.73	0.5	80.4%	600 ($\pm$ 150)
	TLR (B4)	0.64	0.49	0.67	66.5%	300 ($\pm$ 75)
	TL (B4)	0.64	0.74	0.67	62.7%	300 ($\pm$ 75)
	SCR (B4)	0.94	0.37	0.5	70.3%	600 ($\pm$ 150)
	TT (B4)	0.57	0.74	0.31	58.4%	1350 ($\pm$ 450)
LG	TTR (B2)	0.33	0.72	0.5	79.1%	300 ($\pm$ 75)
	TLR (B1)	0.36	0.89	0.62	54.6%	187.5 ($\pm$ 37.5)
	TL (B1)	0.35	0.87	0.62	47.7%	187.5 ($\pm$ 37.5)
	SCR (B2)	0.35	0.87	0.4	33.4%	450 ($\pm$ 150)
	TT (B1)	0.37	0.85	0.44	40.9%	375 ($\pm$ 75)
Sol	TTR (B1)	0.37	0.95	0.62	97.4%	600 ($\pm$ 150)
	TLR (B1)	0.39	0.75	0.76	79.2%	300 ($\pm$ 75)
	TL (B1)	0.35	0.83	0.81	63.1%	225 ($\pm$ 75)
	SCR (B1)	0.36	0.81	0.72	69.8%	375 ($\pm$ 75)
	TT (B1)	0.33	0.98	0.48	63.8%	1050 ($\pm$ 300)
MG	TTR (B2)	0.37	0.86	0.2	73.1%	600 ($\pm$ 150)
	TLR (B3)	0.81	0.53	0.44	22.9%	187.5 ($\pm$ 37.5)
	TL (B2)	0.72	0.66	0.57	37.0%	112.5 ($\pm$ 37.5)
	SCR (B2)	0.72	0.68	0.5	23.5%	150 ($\pm$ 37.5)
	TT (B2)	0.77	0.96	0.1	34.5%	1350 ($\pm$ 450)
