# Supplementary material for: Relevance of selective neural stimulation with a multicontact cuff electrode using multicriteria analysis
Source: PLoS One. 2019 Jul 2;14(7):e0219079. doi: 10.1371/journal.pone.0219079 (PMC6605667; doi:10.1371/journal.pone.0219079)
Supplement: S2 Table — Indexes are presented for each muscle and configuration (with optimal cathode conformation for each muscle). (PDF) [file pone.0219079.s002.pdf]

S2 Table

Muscle	Configuration (Conformation)	SI	Rob	Eff	Recruitment	Intensity (μA) $\pm$ step
TA	TTR (B1)	0.95	0.52	0.55	27.2%	375 ($\pm$75)
	TLR (B2)	0.81	0.43	0.55	27.4%	375 ($\pm$75)
	TL (B1)	0.9	0.47	0.6	29.8%	300 ($\pm$75)
	SCR (B1)	0.78	0.67	0.43	44.6%	600 ($\pm$75)
	TT (B1)	0.76	0.95	0.29	22.8%	1125 ($\pm$375)
LG	TTR (B4)	0.35	0.83	0.5	96.7%	600 ($\pm$75)
	TLR (B1)	0.34	0.86	0.47	88.3%	675 ($\pm$75)
	TL (B2)	0.38	0.83	0.47	78.1%	675 ($\pm$75)
	SCR (B2)	0.36	0.82	0.5	92.2%	600 ($\pm$75)
	TT (B4)	0.37	0.78	0.36	96.2%	1050 ($\pm$150)
Sol	TTR (B4)	0.29	0.88	0.52	76.6%	750 ($\pm$75)
	TLR (B4)	0.3	0.89	0.52	84.7%	750 ($\pm$75)
	TL (B4)	0.33	0.83	0.5	88.0%	825 ($\pm$75)
	SCR (B4)	0.31	0.9	0.52	82.1%	750 ($\pm$75)
	TT (B4)	0.34	0.88	0.48	72.1%	900 ($\pm$150)
MG	TTR (B4)	0.94	0.6	0.45	57.3%	450 ($\pm$75)
	TLR (B4)	0.86	0.32	0.63	56.7%	225 ($\pm$75)
	TL (B3)	0.88	0.42	0.42	49.6%	525 ($\pm$75)
	SCR (B4)	0.85	0.46	0.56	53.1%	300 ($\pm$75)
	TT (B4)	0.82	0.59	0.5	48.1%	375 ($\pm$75)
