# Supplementary material for: Relevance of selective neural stimulation with a multicontact cuff electrode using multicriteria analysis
Source: PLoS One. 2019 Jul 2;14(7):e0219079. doi: 10.1371/journal.pone.0219079 (PMC6605667; doi:10.1371/journal.pone.0219079)
Supplement: S3 Table — Indexes are presented for each muscle and configuration (with optimal cathode conformation for each muscle). (PDF) [file pone.0219079.s003.pdf]

S3 Table

Muscle	Configuration (Conformation)	SI	Rob	Eff	Recruitment	Intensity (μA) $\pm$ step
TA	TTR (B4)	0.7	0.73	0.44	63.1%	375 ($\pm$75)
	TLR (B4)	0.76	0.41	0.62	86.9%	187.5 ($\pm$37.5)
	TL (B4)	0.69	0.51	0.62	65.8%	187.5 ($\pm$37.5)
	SCR (B4)	0.71	0.63	0.62	73.1%	187.5 ($\pm$37.5)
	TT (B4)	0.7	0.98	0.2	92.3%	1200 ($\pm$300)
LG	TTR (B1)	0.36	0.87	0.56	81.2%	600 ($\pm$150)
	TLR (B1)	0.28	0.9	0.56	92.7%	600 ($\pm$150)
	TL (B2)	0.28	0.9	0.67	70.1%	375 ($\pm$75)
	SCR (B2)	0.33	0.84	0.5	74.5%	750 ($\pm$200)
	TT	NAN	NAN	NAN	NAN	NAN
Sol	TTR (B3)	0.83	0.99	0.14	24.3%	225 ($\pm$75)
	TLR (B3)	0.92	0.88	0.25	27.4%	112.5 ($\pm$37.5)
	TL (B3)	0.86	0.99	0.25	28.8%	112.5 ($\pm$37.5)
	SCR (B1)	0.86	0.99	0.5	29.2%	37.5 ($\pm$37.5)
	TT (B2)	0.89	0.97	0.03	30.6%	1050 ($\pm$150)
MG	TTR (B2)	0.83	0.31	0.4	79.9%	450 ($\pm$150)
	TLR (B2)	0.78	0.35	0.57	79.8%	225 ($\pm$75)
	TL (B2)	0.74	0.4	0.5	84.6%	300 ($\pm$75)
	SCR (B2)	0.77	0.4	0.33	75.2%	600 ($\pm$150)
	TT (B2)	0.51	0.75	0.14	35.9%	1800 ($\pm$300)
